# Improved Image Analysis for Measuring Gastric Ulcer Index in Animal Models and Clinical Diagnostic Data

**DOI:** 10.3390/diagnostics12051233

**Published:** 2022-05-14

**Authors:** Chi-Yeol Yoo, Hyeong-U Son, Sung-kook Kim, Si-Oh Kim, Sang-Han Lee

**Affiliations:** 1 Department of Food Science and Biotechnology, Graduate School of Kyungpook National University, Daegu 41566, Korea; yousee0581@knu.ac.kr (C.-Y.Y.); shwcrystal@naver.com (H.-U.S.); 2 Department of Gastroenterology & Hepatology, Kyungpook National University Hospital, Daegu 41944, Korea; skkim@knu.ac.kr; 3 Department of Anesthesiology, Kyungpook National University Chilgok Hospital, Daegu 41404, Korea; sokim@knu.ac.kr

**Keywords:** gastric ulcer, image analysis, ImageJ, conventional clinical observation, ulcer index

## Abstract

Gastric ulcers are one of the most common gastrointestinal diseases. In this study, as an attempt to reduce the minimal error in clinical observations during the diagnosis of gastric ulcers, the applicability of improved ImageJ analysis (IA) was investigated by comparing the results of animal experiments and clinical data. As a result, IA exhibited a significantly improved potential for determining the ulcer index (UI) of clinical data sheets compared to those rated directly by conventional clinical observation (CCO). This indicated that IA enhanced the reproducibility of the measurement of gastric UI using a Bland–Altman plot, resulting in a reduced deviation of each UI value. In addition, it was confirmed that errors in gastric UI decisions can be reduced by adjusting RGB values in diagnostic clinical data (i.e., adjusting to 100 is relatively better than adjusting to 50 or 200). Together, these results suggest that the new enhanced IA could be compatible with novel applications for measuring and evaluating gastric ulcers in clinical settings, meaning that the developed method could be used not only as an auxiliary tool for CCO, but also as a pipeline for ulcer diagnosis.

## 1. Introduction

Gastric ulcers have been established as major gastrointestinal tract diseases which affect 10% of the world’s population to varying degrees, have a high incidence rate, induce serious complications, and are caused by various factors. Gastric ulcers can be caused by *Helicobacter pylori* infection [1], stress, smoking, excessive use of nonsteroidal anti-inflammatory drugs, and excessive alcohol consumption [2]. The most common causal factor is alcohol, which can induce gastric tissue lesions, such as gastritis, gastric ulcers, and even gastric cancer. In vivo studies using ethanol induction, indomethacin administration, and water immersion-restraint (WIRE) stress-induced models have established effective methods to determine the protective effects against gastric lesions [3]. To confirm the expression of the *MUC5* and *MMPs* genes, which protect the gastric mucosa, in vivo experiments were conducted using a mouse model [4,5]. The characteristics of gastric ulcers are well-exposed in the damaged feature of the stomach. Therefore, the state of the stomach is commonly evaluated by an endoscope. In previous studies using a gastric ulcer mouse model, a data-standardized data-scoring technique was used to determine the gastric ulcer index (UI). The gastric UI is well-known for showing standardized scoring criteria, however, it has problems. First, the patterns of ulcer damage caused by artificial means are not the same. Several types of animal models of gastric ulcers, such as ethanol-, indomethacin-, or stress-induced models (including the WIRE model), have been established [6]. The clinical diagnosis revealed that the shapes of the damaged areas are totally different. Second, gastric ulcer scoring is only two-dimensional (gastric ulcer surface). Even if the damaged areas are the same, their depths are not. It is difficult to determine by existing scoring which is more damaged: slight erythema with an extensive area or severe erythema with a small area. In various in vivo experiments, some parts of the indicator cannot be evaluated by a quantitative and automatic instrument, such as a spectrophotometer. For example, although the skin or organ erythema is generally calculated by a caliper [7], this method is possibly affected by the subjectivity of the researcher, leading to low reproducibility and objectivity. To overcome this, analyses with specific software programs, such as ImageJ (a freeware offered from the NIH homepage), photoshop, and MATLAB, are suggested as an alternative method. These computer programs have various benefits with their high accuracy and broad application for biological analysis [8]. Nonetheless, photography has to be supplemented by constantly taking photos with optimized factors, such as ISO value and controlled light source (usually daylight 50~60), because the photo data are not only scientific but also affected by individual differences [9].

However, the ImageJ analysis has been applied for various agricultural, biochemical, and medical purposes. The use of the ImageJ analysis to detect the “woody breast” condition in broiler carcasses has allowed processors to sort broiler carcasses according to woody breast severity among commercial in-line vision grading systems [10]. Furthermore, the ImageJ method is experience-independent, i.e., it can be executed without expert participation in criminological, anthropology, and human anatomy [11]. According to a previous study, by calculating all histological phenotypic parameters—such as mean seminiferous tubule diameter, mean seminiferous lumen diameter, epithelial height, tubular area, and luminal area—by comparing treated and untreated groups using ImageJ, it could be concluded that astaxanthin has beneficial effects [12].

For these reasons, we modified the image analysis to compare the gastric lesions with two types of induced models and clinical diagnostic data. Furthermore, we found that the current method is more efficient, sensitive, and reproducible for calculating the current ulcer index.

## 2. Materials and Methods

### 2.1. Mice and Care

The specific-pathogen-free 7-week-old male BALB/C mice were purchased at 20~22 g of weight from Samtaco Korea (Osan, Korea). The mice were treated under conditions of 12 h of light per day, 22 ± 1 °C temperature, and 50% ± 5% humidity. The mice were allowed to acclimate in the laboratory environment for 1 week prior to the experiment. Animal care was conducted according to the guidelines of the Kyungpook National University Animal Care Committee (approval No. KNU-2014-0069).

### 2.2. Gastric Ulcer Mouse Models

Ethanol was used as an inducer of acute gastric ulcers. The mice were fasted for 24 h before the experiment. All treatment groups were orally administered with 200 μL of 80% ethanol per approximately 25 g of mouse weight (concentration: 8 mL/kg), and the negative control (no treatment (NT) group) group was administrated with saline 30 min before the positive control treatment, as described previously [13,14,15,16,17,18]. The mice were separated into four groups of seven mice each, as follows: NT (no induction of ethanol and saline treatment), saline (ethanol induction and saline treatment), omeprazole (ethanol induction and omeprazole treatment at 3 mg/kg), and gastric sample (ethanol induction and aqueous fraction of *Opuntia humifusa* treatment at 100 mg/kg) groups. For the positive control, 3 mg/kg of omeprazole was orally administrated [13]. Over time, the mice in the treatment groups had drunken symptoms, but no mice died. After 30 min of treatment, euthanasia was performed using carbon dioxide, and the stomach tissues of mice were immediately extracted. The stomach was cut along its greater curvature, and saline was applied to the inner surface area. Then, erythema on the ethanol-induced group (control) was checked, and pictures were taken. For the water immersion-restraint stress (WIRE) model, a previously described protocol with a slight modification was applied to 5 mice per group [3].

### 2.3. General Analysis of Gastric Ulcer

**Figure 1:**
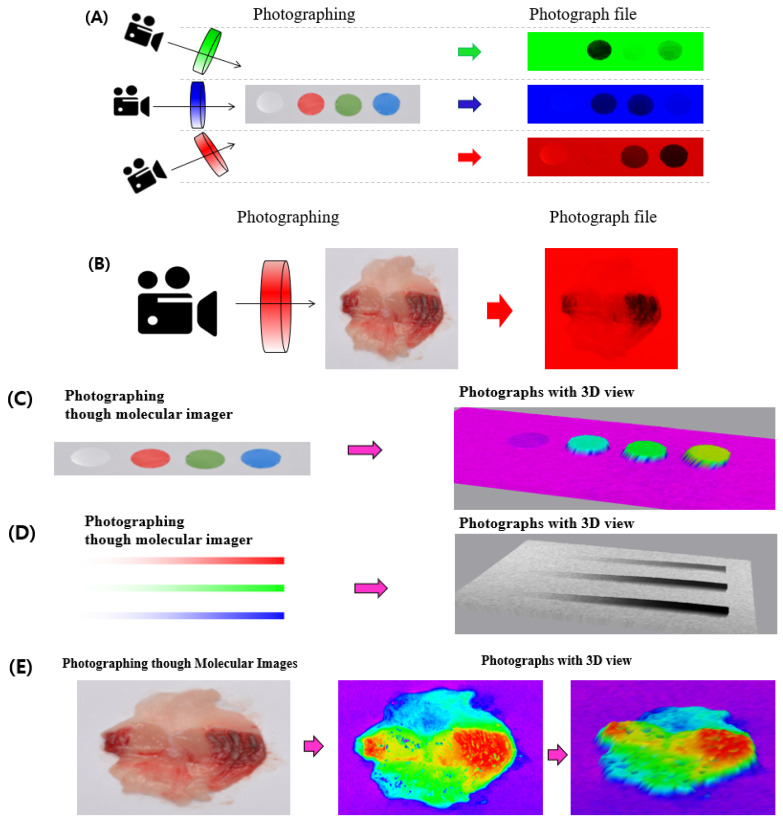
Application of shielding and 3D picture on gastric ulcer image analysis. (**A**) The digital camera was shielded with a cellophane film (green, blue, and red), and the color marker picture was taken. Each color was hidden with the same color shielding after photographing. (**B**) The ethanol-induced gastric organ in a mouse model was photographed with red cellophane film and showed a customized photograph file, which was only determined with a red color. (**C**) The color marker was photographed by a molecular imager. Red, green, and blue without white indicate the height of color density. (**D**) As the color density increases, the height becomes higher in the 3D picture. (**E**) The ethanol-induced erythema damage in a mouse is displayed as a 3D image of the gastric organ.

**Figure 2:**
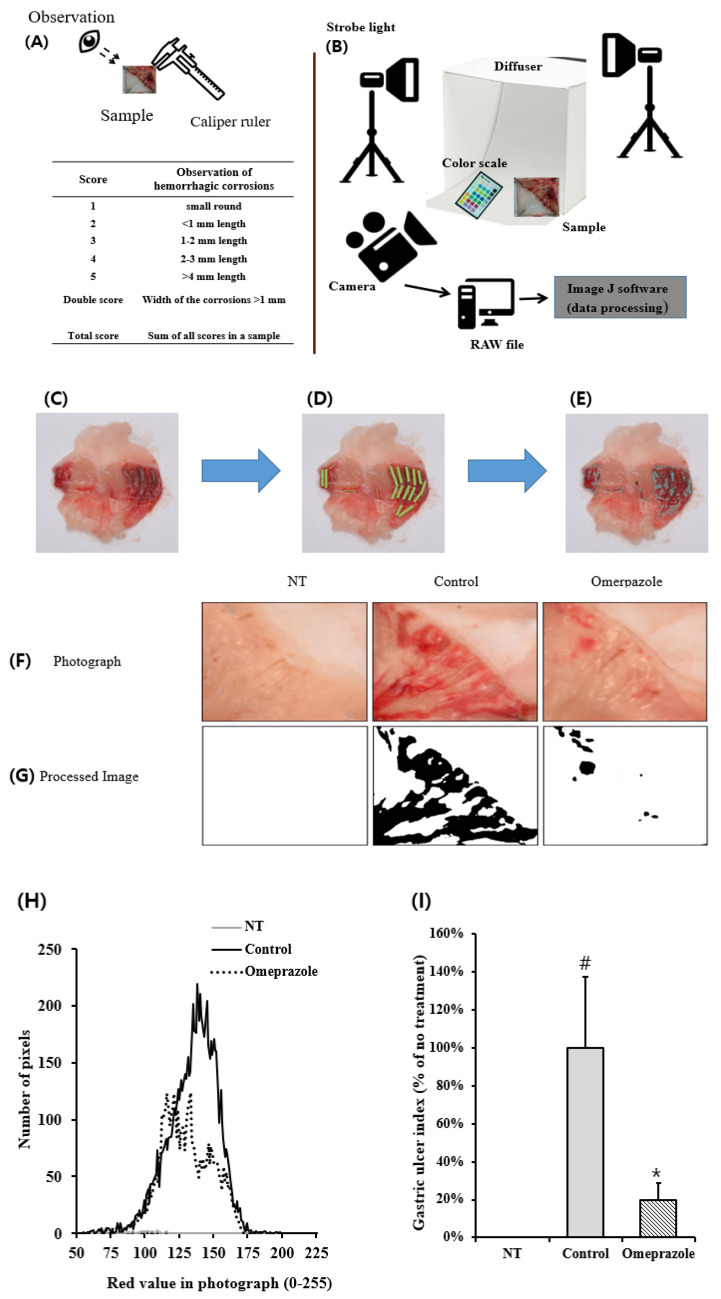
Application of ImageJ analysis on gastric ulcers by comparing the conventional clinical observation (CCO) method. (**A**,**B**) General description of CCO analysis (**A**) and ImageJ analysis (**B**) during determination of the gastric ulcer index. (**C**–**E**) Photographs of ethanol-induced gastric tissue (**C**) are shown to score the gastric ulcer index by CCO (**D**) or by ImageJ analysis (**E**). (**F**) Classical photographs of gastric organ in an ethanol-induced gastric ulcer model. In the model, the damaged zone was classified as slight or severe on the surface of the inner gastric organ by the ImageJ analysis (**G**). (**H**) Histogram of the counted pixel of erythema from the photograph. The pixels were divided according to the red values in the photograph. (**I**) Determination of gastric ulcer index by ImageJ analysis. EtOH, ethanol; Ome, Omeprazole. # *p* < 0.05 and * *p* < 0.01, versus NT (not treated).

To compare the general and modified analysis methods, they were first observed and evaluated using a criterion from previous papers [3,14,15,16,17]. These methods are the same as that of the panel with professional knowledge who observed the inner gastric tissues of the mice using a caliper and scored them based on the criteria (Figure 1 and Figure 2). The criteria for gastric ulceration were: 1 point was assigned for small, round, hemorrhagic corrosions, 2 points were assigned when the length of hemorrhagic corrosions was <1 mm, 3 points were assigned when the length was 1–2 mm, 4 points were assigned when the length was 2–3 mm, and 5 points were assigned when the length was >4 mm. Then, the scores were added and doubled when the width of the corrosions was >1 mm. A panel of eight trained researchers participated to determine the UI, and its general measurement based on the length of the stripe erythema taken by a researcher was considered to be not a quantitative measurement because of subjectivity (Figure 2A). We designated this analysis method to conventional clinical observation (CCO).

### 2.4. Image Analysis of Gastric Ulcer

To take consistent images, the light source and digital raw file were controlled (Figure 2B). To avoid the effect of sunlight and interior lighting, the photos were taken in a dark room, and a strobe (Hyundai Fomax strobe light E400, Seoul, Korea), a Nikon digital camera D5100 (Nikon Co., Tokyo, Japan), and AF-S Micro NIKKOR 40 mm f/2.8 G lens (Nikon Co., Tokyo, Japan) with a Nikon ML-L3 wireless remote control were used. Initially, a color image scale (QP card 203, QP card AB, Helsingborg, Sweden) was photographed after the picture target was placed in a mini studio with a diffuser and background fabric cover [18]. The color image scale is a useful tool to control the color profile for the color character reproducibility and color restoration [19]. Using the same conditions as in the photographed color image scale data, the gastric organs were photographed using the methodology stated in Section 2.2. Gastric ulcer mouse models were placed in the mini studio and photographed. The photographs of the gastric organs were saved as raw files, and their color was adjusted using QP calibration v1.21 software (QP card AB, Helsingborg, Sweden).

### 2.5. Comparison of Optical Measurement and Image Analysis

To evaluate the accuracy of the ImageJ analysis program on visible inflammatory damages, the spectrophotometer and digital camera data information were compared. Since gastric organ damages generally revealed the erythema through gastric mucosal blood flow [20], this value was compared with real blood concentrations. The blood to use a standard erythema was collected from mouse venipuncture. The blood was serially diluted with saline into 50%, 25%, 12.5%, 6.25%, 3.125%, and 0% concentrations in a 6-well plate. A spectrophotometer was used for various assays because of its many advantages, such as high precision, high sensitivity, minimal sample requirements, nonconducting feature, and dynamic range [21]. Therefore, the optical density based on a spectrophotometer at 520 nm was accurate and was used as a standard.

### 2.6. Application into Multidirectional Evaluation via Image Processing

To calculate with minimum background of color information, cellophane films were used (Figure 2A,B). This process corrected the background for green, red, and blue. The digital camera lens was covered with green, blue, or red cellophane films, and pictures were taken under the same condition (Figure 2B).

### 2.7. Image Analysis into Clinical Diagnostic Data

The clinical features were provided from Kyungpook National University Hospital, Korea. Pictures of randomly selected gastric ulcers were used. Their photographs were taken using an Olympus HD260 CCD (charge-coupled device) (Olympus Co., Tokyo, Japan). In total, 140 photographs were analyzed by the ImageJ software. They were assessed by the panel with the same protocol as the mouse model (KNU-2019-0162).

### 2.8. Statistical Analysis

All statistical analyses were performed using SPSS version 20.0 (SPSS Institute, Chicago, IL, USA). The data are presented as means ± standard deviations (SD). Statistical analyses were performed using one-way analysis of variance and Tukey’s test for post hoc comparison. *p*-values < 0.05 were considered statistically significant. To calculate the reproducibility of the analysis methods, a Bland–Altman plot was created [22].

## 3. Results

### 3.1. Application into Multidirectional Evaluation via Image Processing

Some information was excluded from the data analysis as it interrupted the measuring factor in some cases. To analyze the tissues/photograph, large amounts of data were first taken; then, specific information, such as the RGB value, pixel count, or brightness, was selected from the image files. In the analysis of erythema, a low-red value was possibly an interrupting factor as basic organ tissues with no erythematous region also had a red value and can be miscalculated when this region is included in the data. Therefore, covering the low-red value can be a challenge. To cover the RGB information, a cellophane film was used due to its low cost and easy use. The pictures were displayed, blinding each color marker by a green, blue, and red cellophane film in front of the camera lens. The results showed that each blinded color was not exhibited (Figure 1A). Thus, the mouse gastric ulcer tissues with the red cellophane film were adapted, and it was found that the low-red value pixel was unified with the background (Figure 1B). The photograph data of the erythematous lesions were calculated using the ImageJ software, showing that the erythematous lesions had a higher value than the background with the cellophane film.

### 3.2. Measurement of Gastric Ulcer with an Improved Method Using ImageJ Analysis

After euthanasia and opening the inner area of the stomach, severe spots and linear-shaped erythema were observed in the ethanol only group (Figure 2F, second image). However, in the omeprazole-treated group, the gastric lesions in the positive control group were significantly recovered (Figure 2F, third image). To quantitatively determine the gastric ulceration, ImageJ analysis was used to quantify the ulcer index using ImageJ software. First, the photographs were processed, and the damaged zone was divided into white or black (Figure 2G, second and third images). The bisection of the damage degree was adjusted based on the medical doctors’ suggestions or according to experimental differentiation. Thus, the separation of the damaged zone in the photograph (Figure 2G) was easier and more useful than that of the CCO (Figure 2F). The results showed that the ethanol-untreated control group (Figure 2F, first image) rarely indicated the erythematous zone, whereas the ethanol-treated group (Figure 2F, second image) substantially revealed it; however, that of the omeprazole-treated group (Figure 2F, third image) decreased. Second, the pixel number of erythematous zones in the photographs was counted (Figure 2G). Since erythema expresses a red color, only the red value was set as a standard and divided on the x-axis [23]; then, the number of red pixels was counted on the y-axis (Figure 2H). Similar to the photograph data (Figure 2F,G), the ethanol-untreated control group had a low pixel number that indicated erythema, whereas the ethanol-treated group had the highest (Figure 2H). Third, erythematous parts were counted, and gastric UI was determined relative to the ethanol-untreated control group, adjusting it to 100% (Figure 1).

### 3.3. ImageJ Analysis Enhanced the Reproducibility of the Gastric UI Measurement

**Figure 3:**
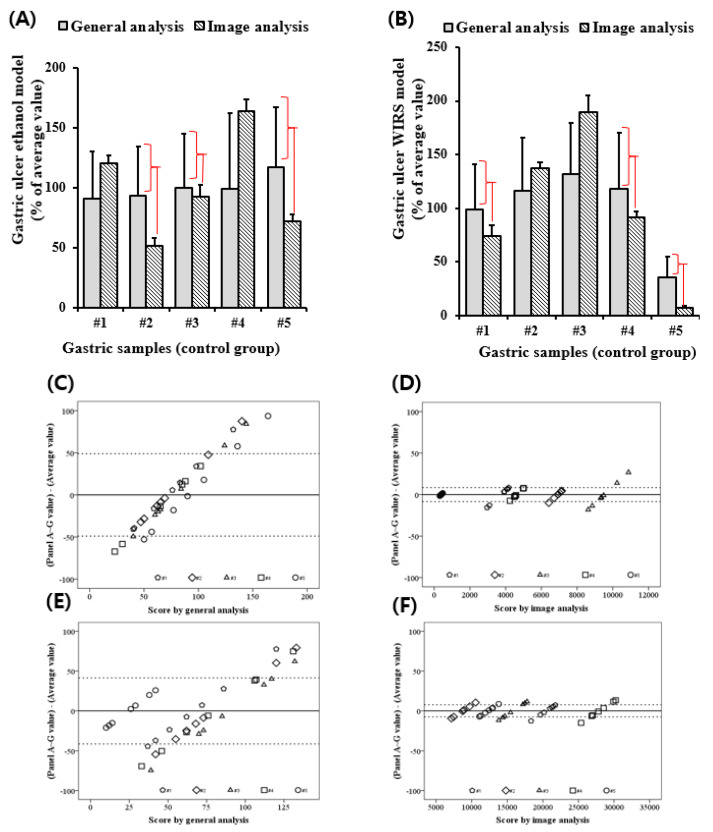
Comparison of gastric ulcer scores observed by CCO and IA. (**A**,**B**) The SD bar indicates the differences among the eight professionals for individual mouse ulcers. To compare the reproducibility according to the same object and different panels, a Bland–Altman plot was created. The y-axis shows the percentage value difference between the professionals and all professionals’ standard deviation. The x-axis shows the gastric ulcer index of CCO (**C**,**E**) and image analysis (**D**,**F**). The lesser deviation (**D**,**E**) of IA means a more reliable gastric UI than that of CCO was retrieved (**C**,**E**). CCO, conventional clinical observation; IA, ImageJ analysis; WIRS, water immersion-restraint stress.

The eye observation analysis (designated as CCO) technique can possibly make a statistical error during gastric UI observation. Thus, it was compared with the ImageJ analysis of gastric UI. Eight trained professionals hand-operated the two methods. Since the ethanol-induced control group was the most damaged and had a high UI, only the ethanol-treated group was compared with all mouse groups. Figure 3A shows the UI of five mice, whereas SD signifies the deviation of the eight professionals. The SD value of the ImageJ analysis was more acceptable than that of the CCO analysis. The Bland–Altman plot, which is a useful statistical analysis used to determine the reproducibility of the experiment, demonstrated that the eye observation had a significantly lower reproducibility than the ImageJ analysis, which also clearly classified the mouse entities (Figure 3C,D). Therefore, it was assumed that the ImageJ analysis was also reasonable for other methods. WIRE was tested in stress-induced mouse models to determine whether the reproducibility of the eye observation and ImageJ analysis had the same pattern in the ethanol model. Since the symptoms of gastric ulcers in the WIRE model, such as the erythematous color, were different than the ethanol model, the technical protocol for using the ImageJ analysis was modified. The ImageJ analysis of the reproducibility of the WIRE model was more important than the CCO of the ethanol model (Figure 3B,E,F).

### 3.4. Determination of the Erythema Value by Image Analysis Using a Blood Standard

**Figure 4:**
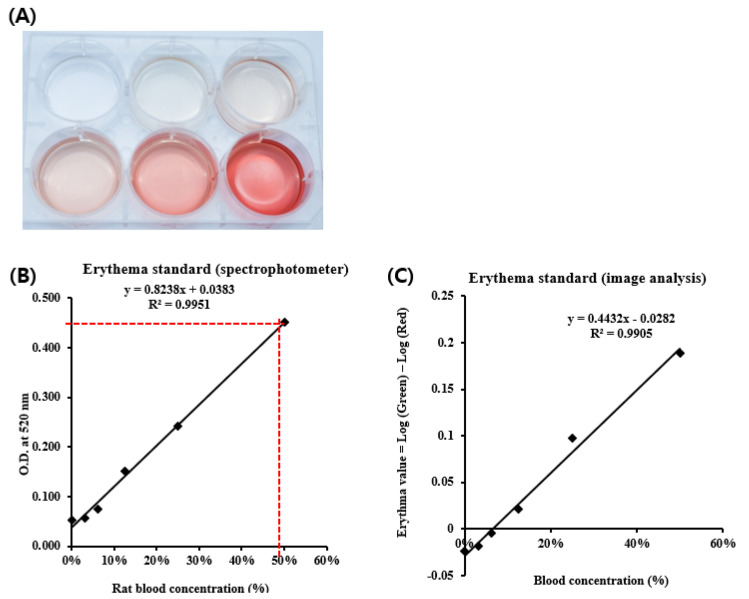
ImageJ analysis using a phased erythema standard. (**A**) Photograph of diluted blood on 6-well plate. The blood was collected from a mouse, and the blood concentrations were 0%, 3.125%, 6.25%, 12.5%, 25%, and 50%. The erythema standard was calculated by spectrophotometer (**B**) and ImageJ analysis (**C**). The erythema range on the photograph was adjusted as a blood.

Since gastric ulcers were revealed with inflammation, severe redness, and mucosal blood flow, their degree was affected by erythematic values, which can be explained by the red color values [24]. To establish a real erythematic value, mouse blood was added to a 6-well plate (Figure 4A) and was measured at 520 nm using a spectrophotometer (Figure 4B). Using a spectrophotometer was a patently elaborate analysis; therefore, the method presented here has the advantage of quantifying the degree and evaluating the distribution of erythema (pigmentation), with minimal interference from pigmentation (erythema). Unlike the color coordinates, such as L*a*b* in the Commission Internationale de l’Eclairage color space, EI and MI are not the indicators in evaluating ‘color’ [25]. The erythema standard was calculated using a spectrophotometer and ImageJ (Figure 4C). Comparing the spectrophotometer and ImageJ analysis of the erythema standard, both methods were fairly precise, with an R^2^ of 0.9951 and 0.9905, respectively (Figure 4B,C). To divide them based on damage degrees, the formula of the erythema standard based on ImageJ analysis (y = 0.4432x − 0.0282) was applied to the gastric tissue photographs and classified as 0–80% of blood-concentrated erythematic values in minimum values. This figure shows that the severe part of erythema, which was also swollen, was balanced at ~60%.

### 3.5. Application in Clinical Diagnosis by Measuring Gastric UI

**Figure 5:**
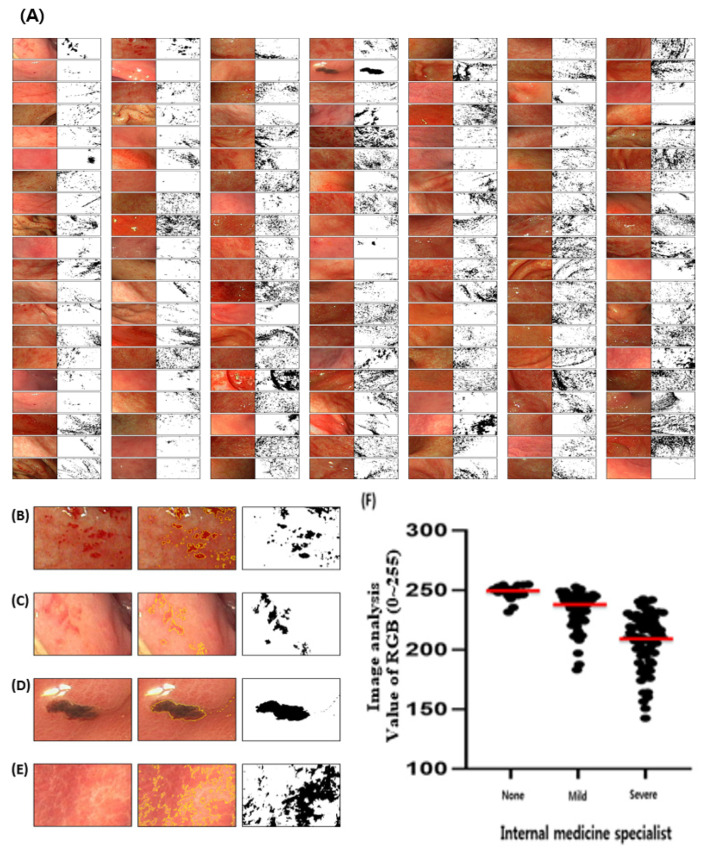
Measurement of gastric ulcers from clinical specimens/images. A 140-image dataset of gastric ulcers was analyzed by the ImageJ software, and a reader study was performed using the 140 images (**A**). Several types of gastric symptoms were observed and classified into severe erythematous area with red spot area (**B**), slight erythema (**C**), *H. pylori* infection (**D**), and defense against ulcer with gastric juice (**E**) as the white area. (**F**) Gastric ulcer measurements in clinical specimens were analyzed with ImageJ by adjusting RGB values between 0 and 225.

**Figure 6:**
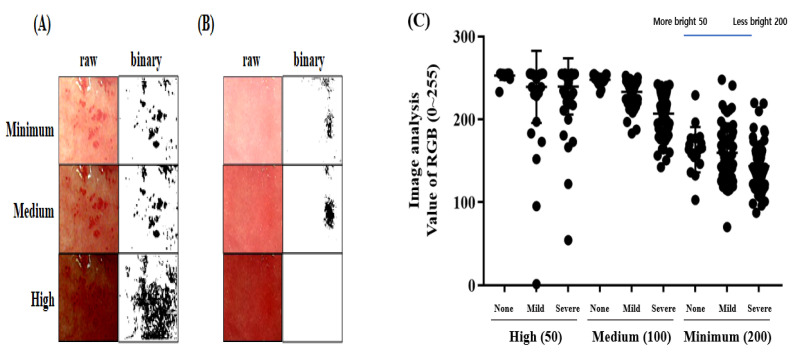
Measurement of gastric ulcers in clinical photographs under different light. The brightness of the images should be optimized enough to identify the symptoms of gastric ulcer/gastritis. In some cases, dark erythema is more clearly distinguished from the normal range as it is brightened (**A**), but light erythema is difficult to distinguish from normal if it is brightened beyond a certain range (**B**). However, when the photo’s light is not sufficient, it is difficult to discriminate the data value regardless of light or dark erythema (**A**,**B**). The gastric ulcer measurements in clinical specimens were analyzed with ImageJ (**C**). The photographs obtained from the first observation were set as the original data, and based on their brightness value, they were classified as high 50, medium 100, and low 200 by setting them as the white balance. The gastric ulcer measurements in clinical specimens were analyzed with different light intensities using ImageJ. The y-axis shows the value of RGB. RGB color has to be maintained between 0 and 255. The x-axis shows the high (50), medium (100), and minimum (200) brightness contrast.

Clinical gastric features were obtained from a 140-image dataset. The clinical diagnosis was determined by selected panels at Kyungpook National University, Korea, and gastric ulcer photographs were classified as alcohol-induced, stress-induced, and drug side effect. Since clinical photographing was not available to control the condition with an exact light and distance, each one’s optional condition was followed on the ImageJ software (Figure 5 and Figure 6). Gastric ulcers were assessed and randomly scored using the ImageJ analysis program for approximately 140 patients (Figure 5A–E). The results exhibited a highly similar pattern to that obtained in a previous ImageJ analysis [26], an AI-based deep learning program, although the model did not match that obtained by gastric ulcer specialists. The model diagnosis gastric ulcers at the same level as residents and provides primary treatment, such as anti-inflammatory drugs. Furthermore, the ImageJ analysis was more effective in diagnosing gastric ulcers when used by an internal medicine doctor alone. These findings indicate that the ImageJ analysis exhibits excellent results compared to those scored directly by the panels that measure gastric UI.

### 3.6. Classification of Gastric Ulcer Lesions by Image Analysis

The degree of gastric ulcers was classified by internal medicine specialists and their values were evaluated through ImageJ analysis. The RGB value of the gastric ulcer pictures was judged as normal, with an average of 247.964. In contrast, the values of mild and severe gastric ulcer pictures judged were 233.184 and 206.857, respectively (Figure 5F). Therefore, the ImageJ analysis value measured according to the diagnosis of gastric ulcers was clearly distinguished. However, it was difficult to accurately define their range with RGB values compared to animal experiments, because the light and camera in the patient’s photographs cannot be consistently controlled (Figure 6A,B). In addition, the results of human gastric endoscopy were more complicated than those of animals. When the amount of light was high, it became easier to observe erythema; however, if the brightness of the picture itself was adjusted after taking it, errors occurred more than in the medium value (Figure 6C). This phenomenon was due to the difference in redness in the normal region. In contrast, it was confirmed that when the brightness was lowered, the erythematous area was broadened as a whole (Figure 6C, high). The data values were erroneously recognized as dark areas instead of erythema because these did not receive even light during shooting. In conclusion, the light intensity should be adjusted to a level where normal/erythematous areas can be distinguished. To obtain a consistent value, it was necessary to implement a constant illuminance. Therefore, in this clinical result, the error in the analysis was because of the unoptimized brightness on some original photographs, and the original data can produce accurate values if the data without errors received constant light in clinical observation. We suggest that making a diagnosis with ImageJ analysis was impossible before supplementation, and we also suggest that this only helps in a clinical setting.

## 4. Discussion

The alleviatory effects of natural and food ingredients against gastric ulcers have been well-documented. Of these, our recent investigation showed that a polymer fraction of *Aloe* *vera* improved the ethanol-induced gastric ulcer based on UI [15]. The gastric UI was determined using the conventional method for scoring and was compared to the normal group. The alleviated group was easily calculable, whereas the severe groups were not calculable due to ambiguous erythematous areas. It is speculated that the calculation time required for CCO is lengthy, and the reproducibility between the observation panels is inconsistent. Therefore, an improved method to determine gastric ulcers was developed using the ImageJ analysis. It was found that the phenotypic ulcer symptoms were unlike those observed when using WIRE- or ethanol-induced mouse models. Red-colored erythematous lesions with consistent lines were observed in ethanol-induced gastric ulcers only, whereas WIRE-induced gastric ulcer symptoms included randomly formed black foreign spots on the inner surface of the mouse stomach [6]. The indomethacin-induced gastric ulcer model also exhibited symptoms similar to those of the ethanol-induced gastric ulcer model according to previous reports [27,28].

Therefore, we investigated whether the ImageJ analysis could be applied to various gastric ulcer-induced experiments. The ethanol model (Figure 3C,D) was relatively effective in inducing gastric ulcers, whereas the WIRE model was not (Figure 3E,F). Optimizing the light brightness and contrast of images derived from the WIRE model proved difficult because erythema appeared deeply during stress induction. The result for the ethanol model was close to the a^*^ value on CIE-Lab, whereas the result for the WIRE model was close to the *L* value, which was influenced by the background color. Moreover, the gene expression in various induction models was different. The indomethacin-treated model showed upregulated EGF, VEGF, myeloperoxidase, and malondialdehyde, and downregulated GSH, GST, SOD, and GPx [29,30], whereas the ethanol-induced model upregulated MMP-9, MMP-2, iNOS, and eNOS [31,32]; moreover, the WIRE-induced model upregulated HSP-70, COX-2, PPAR-γ, NF-κB, ghrelin, and IL-1β, but downregulated PGE_2_ [33,34]. Therefore, the genetic factors of the gastric ulcer induction models seem to differ, although the regulatory factors are similarly related to inflammation, antioxidant potential, and growth factors. Thereafter, it was predicted that these various aspects of gene expression should also be reflected in the phenotypic stomach symptoms. Furthermore, the gastric ulcer measurements in the clinical specimens were analyzed with ImageJ (Figure 5C). The photo brightness of the human gastric ulcer image was set to 100, which was designated as a medium measure. Additionally, relatively high and low values of 50 and 200 were indicated, respectively, as shown in Figure 6. The gastric ulcer measurements of clinical specimens were analyzed with ImageJ at various luminosities. Errors due to false detection of erythema lesions were found in values derived by manipulating the brightness and contrast of the images.

Thus far, ImageJ analysis has been applied to various biochemical data. One method to measure the amplified PCR bands or protein spots observed by antibodies after electrophoresis is taking the quantitative measurements of band strength [35]. In addition, cell counting using an analysis image is simple in mammalian cell cultures [36,37]. Furthermore, because immunohistochemistry (IHC) data are difficult to analyze visually, ImageJ can be used to quantitatively calculate the area of stained cells [38]. The advantages of the ImageJ analysis method against a spectrophotometric technique with image analysis were described earlier. When the graph was shown as a linear equation, the coefficient of determination (R^2^) value validated the data. Therefore, image analysis could be adapted to various diagnoses and predictions of disease symptoms. To accomplish this, a paraffin-embedded tissue section could be observed by hematoxylin and eosin staining or IHC, and computer-aided image analysis would be useful to determine phenotypic characteristics. One related study previously showed that IHC results can be derived from visual scores via the ImageJ analysis [39]. In breast cancer prognosis, the image analysis of the histopathological features of paraffin-embedded breast cancer specimens had a fairly significant effect on the 8-year disease-free survival of 230 patients with breast cancer [40]. Among the 730 features retrieved by the latter study’s clinical results, 4 factors, including the features of tumor nests, tumor nest cell nuclei, and tumor nest cell density, were newly identified as prognostic factors. Meanwhile, bone analysis is usually measured by computed tomography and X-ray microtomography, which are expensive and inflexible. In contrast, the ImageJ image analysis is relatively inexpensive and easy to conduct when analyzing the trabecular bones, whole bones, and osteocyte lacunae [41].

Since the image analysis of gastric ulcers is only suitable for phenotypic data or routine observation, even though it can be applied to IHC, additional analysis from a mechanism study by Western blot or RT-PCR is required. The ImageJ analysis was verified as more rapid, reproducible, and unbiased than CCO, which was affected by the panel’s subjectivity when determining UI. However, for an accurate determination of the image analysis, certain conditions related to the camera background, such as illumination and chromaticity, must be controlled because these can affect the information in the photograph [42]. Specifically, if a large amount of light is present, it becomes easier to observe erythema, but if the brightness of the picture itself is adjusted post-capture (as in this experiment), errors occur more frequently (Figure 6C, minimum). This phenomenon was due to the difference in redness in the normal region. However, it was confirmed that the area recognized as erythema was broadened as a whole when the brightness darkened (Figure 6C, high). Thus, the data values were erroneously recognized as dark areas due to insufficient light during shooting, as with erythema. In conclusion, the intensity of light should be adjusted to a level where normal/erythematous areas can be distinguished. To obtain a consistent value, it is necessary to implement a constant illumination. With such results, it is easier to distinguish whether errors occurred in the analysis because the brightness of some of the original photographs was not optimized. The original data can include accurate values if error-free data, wherein a constant light was received in clinical observation, are secured.

We assumed that it would be difficult to accurately diagnose gastric ulcers without trained experience in clinical practice because the degree and cause of ulceration differ among patients. Accordingly, an auxiliary method that complements the CCO method was found, which could be assessed by clinical experience and by effectively analyzing the photos/images from radiology department computers currently used by clinicians. The reliability of this method was confirmed using the ImageJ software (Figure 1 and Figure 2), and it was confirmed that IA can reduce the deviation of gastric ulcer scores compared with CCO in an animal experiment (Figure 2 and Figure 3). Thus, it might be safe to assume that the deviation of the RGB values in the IA according to the progression of gastric ulcers among the 140 clinical samples could be reduced. To the best of our knowledge, this is the first study to provide scientific evidence that an image analysis can be complementarily applied to the clinical diagnosis of gastric ulcers.

If the limitations of the current method are controlled and improved, the image analysis has the potential to become a beneficial tool for the phenotypic determination of other disease targets in vivo and as a subsidiary for clinical ulcer diagnosis. Although previous studies have demonstrated that image analysis can identify gastric lesions with better diagnostic accuracy than physicians’ clinical observations, the clinical applicability of the method remains uncertain. Further studies are required to demonstrate clinical efficacy in real-world settings to ensure that the improved methodology can be used to determine the intensity of gastric ulcers.

## 5. Patents

Int. Cl. A61B 5/00 (2006. 01) 10-2013-0097146 (KR. B1).

## Data Availability

The data presented in this study are openly available.
